# Ionic Liquid Aqueous Two-Phase Systems for the Enhanced Paper-Based Detection of Transferrin and *Escherichia coli*

**DOI:** 10.3389/fchem.2018.00486

**Published:** 2018-10-16

**Authors:** Matthew F. Yee, Grace N. Emmel, Eric J. Yang, Eumene Lee, Justin H. Paek, Benjamin M. Wu, Daniel T. Kamei

**Affiliations:** ^1^Kamei Laboratory, UCLA, Department of Bioengineering, Los Angeles, CA, United States; ^2^Wu Laboratory, UCLA, Department of Bioengineering, Los Angeles, CA, United States

**Keywords:** ionic liquid, aqueous two-phase systems, lateral-flow immunoassay, transferrin, *Escherichia coli*

## Abstract

Aqueous two-phase systems (ATPSs) have been widely utilized for liquid-liquid extraction and purification of biomolecules, with some studies also demonstrating their capacity as a biomarker concentration technique for use in diagnostic settings. As the limited polarity range of conventional polymer-based ATPSs can restrict their use, ionic liquid (IL)-based ATPSs have been recently proposed as a promising alternative to polymer-based ATPSs, since ILs are regarded as tunable solvents with excellent solvation capabilities for a variety of natural compounds and proteins. This study demonstrates the first application of IL ATPSs to point-of-care diagnostics. ATPSs consisting of 1-butyl-3-methylimidazolium tetrafluoroborate ([Bmim][BF_4_]) and sodium phosphate salt were utilized to quickly concentrate biomarkers prior to detection using the lateral-flow immunoassay (LFA). We found the phase separation speed of the IL ATPS to be very rapid and a significant improvement upon the separation speed of both polymer-salt and micellar ATPSs. This system was successfully applied to both sandwich and competitive LFA formats and enhanced the detection of both *Escherichia coli* bacteria and the transferrin protein up to 8- and 20-fold, respectively. This system's compatibility with a broad range of biomolecules, rapid phase separation speed, and tunability suggest wide applicability for a large range of different antigens and biomarkers.

## Introduction

While global health has improved over the last few decades, health pandemics in resource-poor settings remain a large problem (Scarborough and Thwaites, 2008; Tang and Nour, 2010; Gunasekera and Pathiraja, 2016). These health issues include chronic health conditions, such as diabetes (Nugent, 2008) and infectious diseases, such as tuberculosis (Global Tuberculosis Report 2017, 2017). In countries like the U.S, many of these illnesses are readily treatable, especially when diagnosed early; however, in resource-poor settings, patients lack easy access to standard laboratory-based tests such as the enzyme-linked immunosorbent assay (ELISA), nucleic acid amplification tests, and serology tests (Elbireer et al., 2011). With issues such as poor infrastructure and limited funding already leading to underutilization of central laboratories in these resource-poor settings (Elbireer et al., 2011; Nkengasong et al., 2018), there is a growing interest in developing point-of-care techniques to diagnose a variety of diseases. Devices, such as miniaturized bioelectronics and microfluidic tests like the lateral-flow immunoassay (LFA), have received much attention over recent years due to their ease-of-use, portability, and limited need for power (Li et al., 2015; Sharma et al., 2015; Gumustas et al., 2018; Wang et al., 2018). However, in comparison to the gold standard laboratory-based tests, these devices are still restricted by their limited sensitivity, indicating an increasing need for enhanced detection capabilities at the point-of-care.

One technique for enhancing point-of-care detection is the aqueous two-phase system (ATPS), a liquid-liquid extraction system that has previously been demonstrated to concentrate biological markers (Hatti-Kaul, 2001). ATPSs consist of two immiscible phases, similar to oil-water systems; however, both phases of an ATPS are aqueous-based. Molecules introduced into an ATPS can experience extreme partitioning between the two phases based on the excluded-volume, hydrophobic, and electrostatic interactions they experience with the components of each of the two phases. Furthermore, ATPSs are much more biocompatible than conventional oil-water systems, and have been widely utilized for the purification of proteins and nucleic acids (Hatti-Kaul, 2001). While the ATPS has been traditionally used in large-scale, industrial bioseparations, it also lends itself well for applications in point-of-care settings, as it is easy-to-use, can be rapid, and is scalable (to minimize sample volume) (Iqbal et al., 2016). In addition, ATPSs do not require laboratory equipment and are low in cost compared to more conventional laboratory tests such as the ELISA and nucleic acid amplification.

For these reasons, our research group has recently demonstrated the use of the ATPS as a pre-concentration tool to improve the sensitivity of portable paper-based diagnostic tools such as the LFA. Through the use of conventional polymer-salt and micellar systems, ATPSs combined with existing detection technologies have been shown to enhance sensitivity in detecting various biomarkers, including 10-fold improvements in LFA detection of parasitic biomarkers (Pereira et al., 2015) and viruses (Mashayekhi et al., 2010). In a similar manner, the ATPS was also shown to improve sensitivity and decrease time-to-detection of a paper-based spot immunoassay (Cheung et al., 2017). However, despite the efficacy of these systems, the limited polarity range of these ATPSs can restrict their use (Freire et al., 2012), particularly regarding the partitioning of small hydrophilic proteins. As micellar and polymer ATPSs predominantly rely on excluded-volume interactions to partition hydrophilic biomolecules to a particular phase, smaller hydrophilic biomarkers such as proteins can be difficult to partition extremely. One approach to improve upon this issue is to fine-tune the polarity of the ATPS components and introduce electrostatic effects as a more significant factor in partitioning.

One potential solution is through the use of ionic liquids (ILs), which are salts that are molten at low temperatures. ILs have been investigated as alternatives to traditional, volatile organic solvents as they exhibit non-flammability and negligible volatility (Freire et al., 2012) due to their ionic nature. In addition, they are particularly promising for use in an ATPS as they are highly tunable and have excellent solvation capabilities (Berthod et al., 2008) for a variety of natural compounds and proteins. This has led to their use in various extraction and separation processes (Liu et al., 2007; Berthod et al., 2008; Tang et al., 2012) including ATPSs. These systems were found to phase separate with the mixture of kosmotropic salts and imidazolium-based ILs (Gutowski et al., 2003). Since then, different classes of ILs have been discovered, developed, and utilized in the formation of ATPSs (Wilkes et al., 1982; Ventura et al., 2011); this variety in ILs, combined with an even greater variety in salts, could potentially allow researchers to precisely concentrate smaller biomolecules that would otherwise be difficult to partition extremely into one phase through excluded-volume interactions alone.

One of the most commonly investigated types of IL ATPSs are imidazolium-based. This class of ILs has shown promising potential as an extraction technique for a wide variety of compounds, including proteins, amino acids, and antibiotics (Freire et al., 2012). Additionally, these systems are optimal for point-of-care diagnostics since they are able to phase separate at room temperature and at physiological pH. In this study, IL ATPSs consisting of 1-butyl-3-methylimidazolium tetrafluoroborate ([Bmim][BF_4_]) and sodium phosphate salt were utilized to demonstrate the compatibility of the IL ATPS with LFA and the ability of this technique to improve the sensitivity of LFA tests. This enhancement was applied to the model protein transferrin, using the competitive LFA format, and the model pathogen *Escherichia coli* O157:H7, using the sandwich LFA format. To our knowledge, this is the first application of an IL ATPS for the enhancement of point-of-care diagnostics. The IL ATPS demonstrated very fast phase separation and was found to be directly compatible with LFA, requiring no additional modification to existing LFA structure; by utilizing these benefits and also a significant enhancement effect, our system addresses limitations faced by existing paper-based portable diagnostics regarding the concentration of small biomarkers.

## Materials and methods

### Preparation of bacterial cell cultures

*Escherichia coli* O157:H7 bacteria (*E. coli*) (ATCC® 700728™) were grown and cultured according to manufacturer protocol (ATCC, Manassas, VA) and plated onto Difco Nutrient Agar (Becton, Dickinson and Company, Sparks, MD) plates. Plated cells were subsequently incubated at 37°C aerobically overnight. The incubated plates were then sealed with Parafilm and stored at 4°C until use. To prepare bacterial suspensions for use in ATPS and LFA tests, single colonies were picked from the agar plate and cultured in 5 mL of Difco Nutrient Broth (Becton, Dickinson and Company, Sparks, MD). The cell suspension was then incubated in a shaker-incubator at 37°C and 200 rpm for 16 h. After use in LFA tests, the concentrations of bacteria in the suspensions were determined through plating of bacteria following serial dilutions. These bacteria were then incubated at 37°C aerobically overnight, after which the colonies were counted in order to quantify the bacterial concentrations used in the tests.

### Preparation and visualization of IL ATPSs

Compositions of IL and salt necessary to achieve the desired equilibrium volume ratios, i.e., the volume of the top phase divided by the volume of the bottom phase, were determined by varying the initial concentrations of both [Bmim][BF_4_] (Sigma-Aldritch, St. Louis, MO) and sodium phosphate (2:1 dibasic:monobasic) in solutions of Dulbecco's phosphate-buffered saline (PBS; Invitrogen, Grand Island, NY, pH 7.4). Conditions for 1:1 and 1:9 volume ratio ATPSs were found to be 35% w/w [Bmim][BF_4_] and 3% w/w salt, and 65% w/w [Bmim][BF_4_] and 0.5% w/w salt, respectively. Additionally, 0.01% w/w Triton X-100 surfactant (Sigma-Aldritch, St. Louis, MO) was added to the 1:9 IL ATPS to facilitate phase formation. These conditions were used for all of the following experiments.

For visualization of phase formation in the IL ATPSs, 44 or 8.8 μL of bovine serum albumin-coated dextran-coated gold nanoparticles were added to 1.5 g 1:1 volume ratio or 1:9 volume ratio ATPSs, respectively. These ATPSs were well-mixed to ensure a homogenous mixture. The ATPSs were then incubated at room temperature. Time-to-equilibrium was established when the visible domains arrived at their respective macroscopic phases, and the location of the interface remained stable.

### Viscosity and interfacial tension measurements

Viscosity and interfacial tension measurements were performed using 30 g of IL-, polymer-, and micelle-based 1:1 volume ratio ATPSs. IL-based ATPSs were prepared as previously mentioned. Polymer-based ATPSs consisted of 12.5% w/w PEG 20k (Sigma-Aldritch, St. Louis, MO) and 7.5% w/w potassium phosphate (5:1 di:monobasic) in solutions of PBS. Micelle-based ATPSs consisted of 4% w/w Triton X-114 (Sigma-Aldritch, St. Louis, MO) in solutions of PBS.

Du Noüy ring interfacial tension measurements for each ATPS were obtained utilizing the Krüss K6 force tensiometer (Krüss USA, NC, USA). A standard platinum ring attached to the tensiometer was used in the measurements. Viscosity measurements were obtained using the Brookfield LVDV-I Prime digital viscometer (AMETEK Brookfield, MA, USA). All tests were performed at room temperature. Triplicate measurements of each condition were performed.

### Detection of transferrin (Tf)

#### Preparation of dextran-coated gold nanoprobes (DGNPs)

Purple colored dextran-coated gold nanoparticles (DGNs) were synthesized according to Min and coworkers with slight modifications (Jang et al., 2013; Chiu et al., 2014). Briefly, 0.75 g of dextran (M_w_ 15000–25000) were dissolved in 9.9 mL of filtered UltraPure sterile water (Rockland Immunochemicals Inc., Gilbertsville PA). The solution was stirred and heated to a boil, after which 135 μL of 1% w/v gold (III) chloride hydrate were added. The color of the reaction mixture became dark purple, and the solution was stirred and boiled for 20 more minutes. The particles were stored at 4°C until use.

The dextran-coated gold nanoprobes (DGNPs) were prepared as follows. A 1 mL aliquot of dextran-coated gold nanoparticles was adjusted to pH 9.0 using 0.5 M NaOH. Subsequently, 4 μg of anti-transferrin (anti-Tf) antibodies were added to the solution. The mixture was placed on a shaker for 30 min to facilitate the formation of dative bonds between the antibodies and the dextran-coated gold nanoparticles. Free antibodies were removed by centrifugation. The pellet was resuspended in 100 μL of 0.1 M of sodium borate buffer at pH 9.0.

#### Preparation of competitive LFA strips

**Figure 1:**
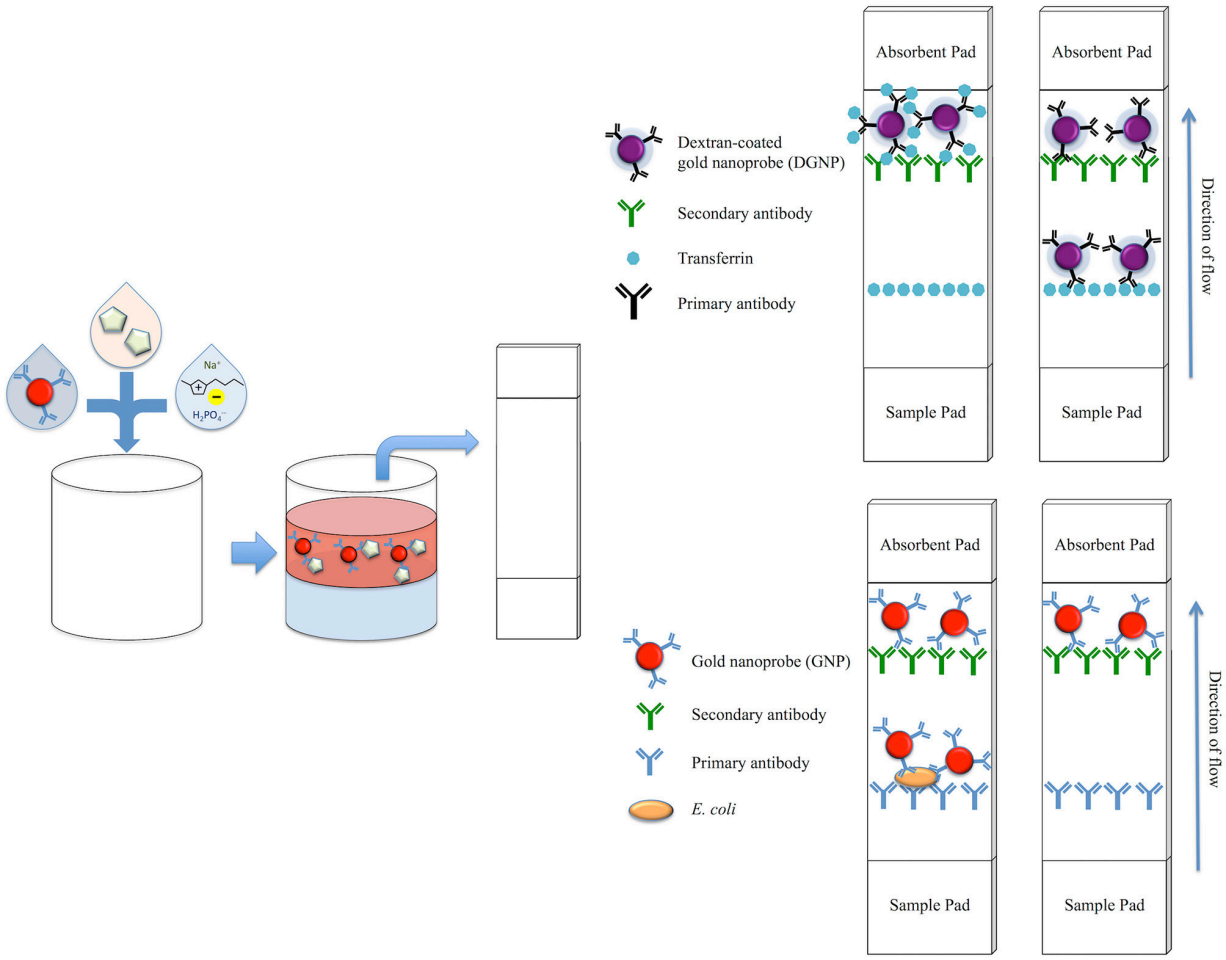
Schematic of biomarker concentration with an IL ATPS and subsequent detection on the LFA. Gold nanoprobes and phase-forming components are first mixed with a known concentration of target analyte. Following phase separation, the top phase is extracted and applied to both the competitive format LFAs for detection of transferrin and sandwich format LFAs for the detection of *E. coli*. For the competitive format LFA, which utilizes purple-colored DGNPs, a positive test is indicated by a single purple band, while a negative test is indicated by two purple bands. For the sandwich format LFA, which utilizes red-colored GNPs, a positive test is indicated by two red bands, while a negative test is indicated by one red band.

LFA test strips utilizing the competitive assay format were assembled in a similar manner to our previous studies (Mashayekhi et al., 2012). Briefly, both Tf and rabbit anti-goat IgG secondary antibodies were dissolved in a 25% w/v sucrose solution for stabilization prior to being printed in lines on a CN140 nitrocellulose membrane. After drying overnight, the nitrocellulose was treated with 1% w/v BSA to prevent nonspecific binding. Subsequently, the 1% w/v BSA treated S17 fiberglass sample pad, the nitrocellulose membrane, and CF4 absorbent pad were assembled onto an adhesive backing into 5 mm wide LFA strips. In this format, immobilized Tf constitutes the test line and immobilized secondary antibodies specific to the primary anti-Tf antibody constitute the control line. If enough Tf is present to saturate the antibodies immobilized to the DGNPs in a sample, the Tf-DGNP complexes flowing through the LFA strip will not bind to the immobilized Tf on the test line. This results in the absence of a visible purple band at the test line region. If Tf is not present, unbound antibodies on the DGNPs will bind to the immobilized Tf, and a visual band will form at the test line. In either case, the antibodies on the DGNPs will bind the secondary antibodies immobilized at the control line and form a visible line, indicating successful sample flow through the strip. Therefore, a positive result is indicated by only one purple band at the control line, while a negative result is indicated by two purple bands at both the test line and control line (Figure 1).

#### Detection of Tf with LFA only

To verify the detection limit of Tf with LFA only tests, anti-Tf DGNPs were added to a sample solution in a test tube and allowed to bind to Tf present in the sample to form Tf-DGNP complexes. Tests consisted of 50 μL sample solution, which was composed of 3 μL of anti-transferrin DGNPs and 47 μL of a known amount of Tf dissolved in PBS, or only PBS for the negative control. The solution was incubated for 10 min at room temperature to allow the DGNPs to capture the Tf in solution. The LFA test strip was then inserted vertically into the sample solution, which wicked through the strip via capillary action toward the absorbent pad. Images of the test strips were taken immediately after 20 min with a Nikon D3400 camera in a controlled lighting environment. Triplicates of each test were obtained and analyzed with a custom MATLAB program.

#### Detection of Tf with the IL ATPS/LFA setup

For detection of Tf with the 1:1 volume ratio ATPS, 120 mg of a well-mixed 1:1 ATPS containing 3.6 μL of anti-Tf DGNPs and a known concentration of Tf were added into a test tube. The solution was incubated for 10 min at room temperature to allow the DGNPs to capture the Tf in solution and to allow the ATPS to phase separate. The top phase was extracted and placed in a new test tube, and the LFA test strip was inserted vertically into the sample solution as explained previously (Figure 1). For detection of Tf with the 1:9 ATPS, 600 mg of a well-mixed 1:9 volume ratio ATPS containing 4.8 μL of anti-Tf DGNPs and a known concentration of Tf were added into a test tube. These overall ATPS volumes were chosen to maintain the sample volume applied to the LFA at 50 μL. The remainder of the procedure follows the methods outlined for detection with the 1:1 ATPS. Images of the test strips were taken immediately after 20 min with a Nikon D3400 camera in a controlled lighting environment. Triplicates of each test were obtained and also analyzed with a custom MATLAB program.

### Detection of *E. coli*

#### Preparation of gold nanoprobes (GNPs)

Cherry-colored gold nanoparticles of diameter 40 nm (Nanocomposix, San Diego, CA) were obtained and stored at 4°C until use. To prepare functional probes for use in the LFA tests, the pH of the gold nanoparticle solution was adjusted to pH 9.0 using 0.5 M NaOH. For every 1 mL of gold nanoparticle solution, 8 μg of anti-*E. coli* antibodies were added. The reaction mixture was placed on a shaker for 30 min to facilitate formation of dative bonds between the antibodies and gold nanoparticles. Free antibodies were removed through centrifugation. The pellet was resuspended in 100 μL of 0.1 M sodium borate buffer at pH 9.0 and subsequently stored at 4°C until use.

#### Preparation of sandwich LFA strips

LFA test strips utilizing the sandwich style assay were prepared in a similar manner to our previous studies (Mashayekhi et al., 2010). Briefly, both primary anti-*E. coli* antibodies and rabbit anti-goat IgG secondary antibodies were dissolved in a 25% sucrose solution for stabilization prior to being printed in lines on the CN95 nitrocellulose membrane. After overnight drying, the membrane was treated with 1% BSA. Subsequently, the 1% BSA treated S17 fiberglass sample pad, the nitrocellulose membrane, and CF4 absorbent pad were assembled onto an adhesive backing into 5 mm wide LFA strips. For the sandwich style format, anti-*E. coli* antibodies specific for the target *E. coli* are immobilized at the test line, while secondary antibodies against the primary anti-*E. coli* antibody are immobilized at the control line. If enough *E. coli* is present in the sample, *E. coli* will bind to the antibodies on the GNPs, producing *E. coli*-GNP complexes. These will bind to primary antibodies on the test line, trapping the particles and forming a visual red band. Alternatively, if the target biomarker is not present, the colloidal gold will bypass the test line without binding. Regardless, antibodies immobilized on the GNPs will bind the secondary antibodies on the control line, forming a visual band and therefore indicating a valid test. Thus, the presence of one line at the control line indicates a negative test, while the presence of two lines at both the control line and test line indicates a positive test (Figure 1).

#### Detection of *E. coli* with LFA only

Tests with LFA only were performed as now described. Solutions containing *E. coli* suspensions in Nutrient Broth were first prepared, with *E. coli* concentrations serially diluted from an initially prepared culture in Nutrient Broth to achieve a range of concentrations for detection. Five microliters of diluted *E. coli* suspension, or 5 μL of pure Nutrient Broth for the negative control, were added to 40 μL of PBS and 5 μL of anti-*E.coli* GNPs for a constant sample volume of 50 μL. The resulting solutions were mixed and incubated for 10 min to allow for binding between *E. coli* and the GNPs. A test strip was dipped vertically into each solution, and the sample was allowed to wick up the LFA. After 20 min, the LFA strips were taken out of the solution, and an image of each strip was immediately taken with a Nikon D3400 camera in a controlled lighting environment. Triplicates of each test were obtained; images were analyzed visually and quantified using a custom MATLAB program.

#### Detection of *E. coli* with the IL ATPS/LFA setup

For tests combining the ATPS with LFA, 120 mg of a 1:1 ATPS containing 5 μL GNPs and 12 mg of an *E. coli* suspension were added to a test tube. The suspension was incubated for 10 min, after which the top phase was extracted and tested as described previously for the LFA only tests. For the 1:9 ATPS, 600 mg of an ATPS containing 5 μL GNPs and 60 mg of an *E. coli* suspension were added to a tube and tested in a similar manner to the 1:1 ATPS runs. These overall volumes were chosen to maintain the sample volume applied to the LFA at 50 μL. The tests were also run for 20 min and immediately imaged with a Nikon D3400 camera in a controlled lighting environment. Triplicates of each test were obtained, and the images were analyzed via a custom MATLAB program.

### LFA quantification

A custom MATLAB script was written with an approach similar to Yager and coworkers (Fu et al., 2011) to quantitatively analyze the LFA tests. Images of the test strips were taken with a Nikon D3400 camera under controlled lighting, with each strip oriented the same way. The images were cropped and converted to an 8-bit grayscale matrix. The intensity was averaged along the axis perpendicular to the flow, and therefore parallel to both the control and test lines, generating a one-dimensional intensity map. The two maxima were identified as the control and test lines, with the distance between the two lines calibrated by using a reference LFA image with strong test and control lines. In the case of the transferrin competitive assay, this corresponded to the negative control, and in the case of the *E. coli* sandwich assay, this corresponded to the positive control.

To obtain test line intensity from our sample data, the location of the control line was determined from the reference LFA image, and its distance from the test line was calibrated as described above. The test line region was set as a 15 pixel-wide region centered at this location. The baseline for the measurement was determined by averaging the signal from two 25 pixel wide boxes beginning 25 pixels before and 25 pixels after the center of our determined test line region. The test line intensity was then calculated as the area under the curve for this test line region.

## Results

### Visualization of IL ATPS phase separation

Several criteria were used to determine a suitable ATPS for use in this study. Our anticipated design involved taking advantage of rapid phase separation speeds of IL-based systems to avoid issues faced using polymer or micellar systems. To achieve ease-of-use and minimize extra user handling steps, we sought an ATPS where our probes would partition to the top phase. Additionally, maintaining physiological pH and low ionic content in the phase DGNs and GNs partition preferentially to (i.e., the salt-rich phase) were considered, to preserve antibody function for use in the LFAs. With these considerations, [Bmim][BF_4_] and sodium phosphate salt (2:1 dibasic:monobasic) were chosen as the components of the ATPS. These components successfully phase separated, and allowed for relatively low salt concentrations as well as a pH of 7.0 in the top phase, which was optimal for our applications. A schematic of the IL ATPS, along with competitive and sandwich LFA formats, can be found in Figure 1.

**Figure 2:**
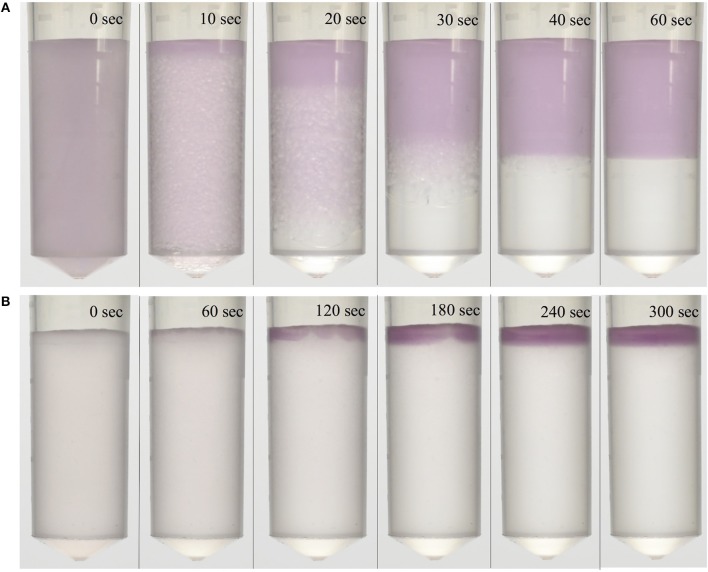
Visualization of phase separation and speed of an IL ATPS. Purple-colored dextran-coated gold nanoparticles partitioned extremely to the top phase and were used to visualize separation. ATPSs separated within **(A)** 1 min for the 1:1 ATPS, and **(B)** 5 min for the 1:9 ATPS.

1:1 and 1:9 volume ratio ATPSs were achieved, and phase separation was visualized through the addition of bovine serum albumin-coated DGNs. The DGNs partitioned preferentially into the top phase, indicated by the purple-colored top phase, while the bottom phase remained clear due to the absence of DGNs. The particles were found to be stable in the ATPS, exhibiting no signs of aggregation over several days. These visualization experiments were also performed utilizing bovine serum albumin-coated gold nanoparticles (GNs), which exhibited similar partitioning and stability behavior. In all cases, phase separation was found to be quite rapid, occurring in 1 min for the 1:1 ATPS (Figure 2A) and in 5 min for the 1:9 ATPS (Figure 2B). As 9:1 polymer-salt ATPSs can take an hour or so to phase separate, and 1:9 micellar systems even longer, this marked a great improvement in phase separation time compared to conventional ATPSs.

### Viscosity and interfacial measurements

The viscosities of the top and bottom phases of 1:1 volume ratio IL-, polymer-, and micelle-based ATPSs were measured, as shown in Table 1. While salt-rich and micelle-poor phase viscosities were comparable to one another for the three ATPSs, the IL-rich phase viscosity was significantly lower than those of the micelle-rich and polymer-rich phases.

**Table 1:** Viscosity measurements for the phases of 1:1 volume ratio IL-, polymer-, and micelle-based ATPSs.

**Type of ATPS**	**Type of phase**	**Viscosity (cP)**
Polymer	Salt-rich	1.83 ± 0.03
	Polymer-rich	23.9 ± 1.3
Micellar	Micelle-poor	1.55 ± 0.04
	Micelle-rich	120.7 ± 0.5
Ionic Liquid	Salt-rich	1.95 ± 0.02
	Ionic liquid-rich	4.1 ± 0.1

*The viscosity of the IL-rich phase was much lower than the viscosities of the polymer-rich and micelle-rich phases investigated*.

Additionally, interfacial tension measurements were performed for the IL-, polymer-, and micellar-based ATPSs. The interfacial tension of the 1:1 volume ratio IL ATPS was found to be 1.8 ± 0.3 $\frac{mN}{m}$. The interfacial tensions for the polymer- and micelle-based ATPSs were below the equipment's lower limit of detection of 1 $\frac{mN}{m}$. This is consistent with reports in the literature of interfacial tensions of polymer-based ATPSs being in the range of 0.1–1.9 $\frac{mN}{m}$ depending on their composition (Wu et al., 1996). Accordingly, the interfacial tension of the IL ATPS was significantly higher than the polymer- and micelle-based ATPSs that we have studied.

### Detection of Tf

Upon identifying compositions for 1:1 and 1:9 IL ATPSs, we investigated the degree of improvement in utilizing these ATPSs for the detection of the model biomarker Tf. To do this, we sought to determine the limit of detection of Tf utilizing an LFA-only set-up, and then compare it directly with the limit of detection obtained utilizing the IL ATPS/LFA setup. As previously mentioned, these experiments were performed as competitive assays; two bands would indicate a negative test, as the antibodies on the DGNPs would be able to bind the immobilized Tf at the test line, and a single band at the control line would indicate a positive test, as DGNP antibody binding sites would be saturated and therefore unable to bind the Tf on the test line. With these mechanisms in mind, the limit of detection was defined as the lowest concentration of Tf at which the test line was not visible.

**Figure 3:**
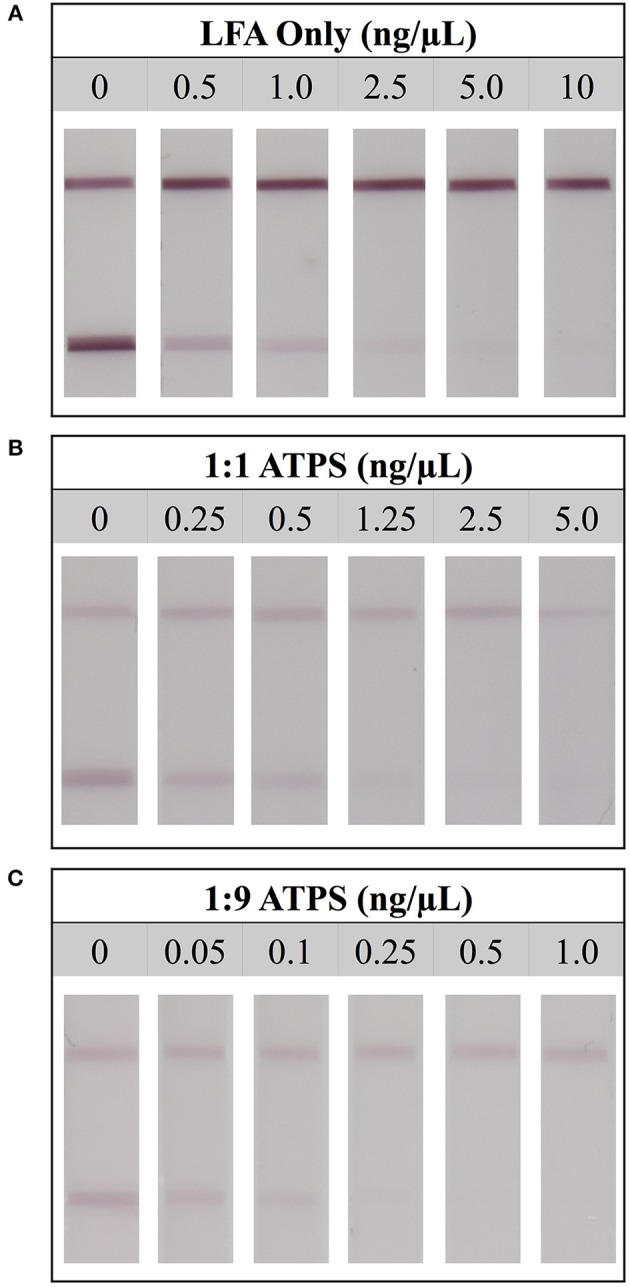
Limit of detection for LFA tests detecting for protein transferrin. **(A)** Detection limit for the LFA-only test was found to be 5 ng/μL. **(B)** Detection limit for the LFA when combined with a 1:1 ATPS was 1.25 ng/μL, and **(C)** detection limit for the LFA when combined with a 1:9 ATPS was 0.25 ng/μL, indicating 4-fold and 20-fold improvements, respectively.

**Figure 4:**
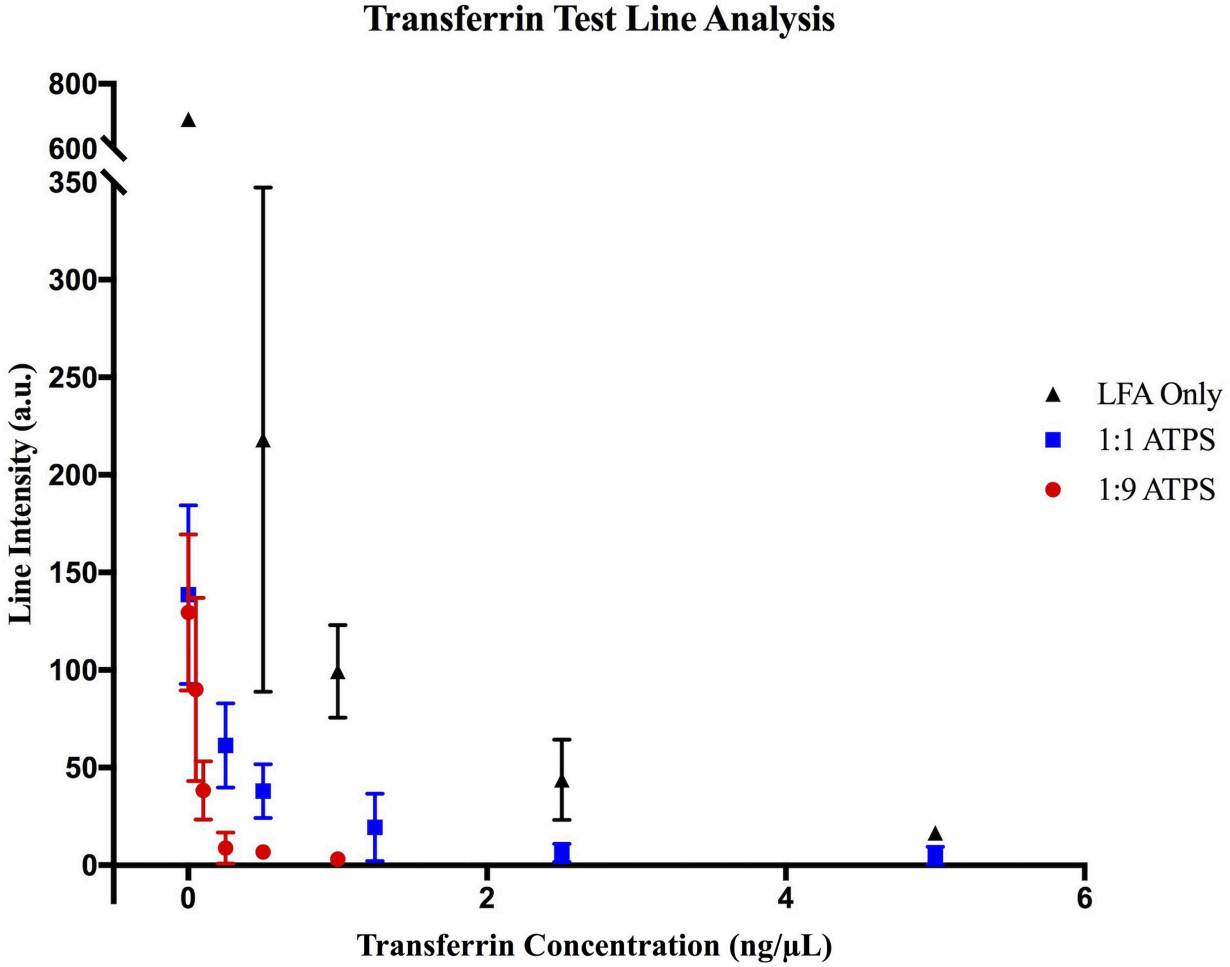
MATLAB analysis of transferrin LFA tests. Data for (▴) LFA-only tests without ATPS enhancement, (■) enhancement with a 1:1 ATPS, and (●) enhancement with a 1:9 ATPS. At each concentration, test line intensities in arbitrary units (a.u.) were lower for the 1:1 ATPS/LFA test than the LFA-only test and lower for the 1:9 ATPS/LFA test than the 1:1 ATPS/LFA test, indicating improved detection with more extreme volume ratios.

For the LFA-only tests, when a high concentration of Tf was used (10 ng/μL), test lines did not develop, indicating a true positive result. However, at a lower concentration (2.5 ng/μL), a test line was present, indicating a false negative result. This suggested that the limit of detection for LFA without ATPS enhancement was 5 ng/μL (Figure 3A). A similar analysis was performed for the 1:1 and 1:9 ATPS setups and the limits of detection were found to be 1.25 ng/μL (Figure 3B) and 0.25 ng/μL (Figure 3C), respectively, indicating 4- and 20-fold improvements in detection over LFA-only. The improvement was significant, but the test lines were fainter compared to the LFA only tests. The control line intensities were also fainter. These findings were confirmed via our MATLAB analysis; test lines were less developed across all concentrations, including our negative control at 0 ng/μL (Figure 4). We also observed a large standard deviation in the test line intensity of LFA only for 0.5 ng/μL, which is likely due to variability in the background signal. However, this variability did not have a significant effect on our conclusions from the MATLAB analysis, as the error bars for different tests did not overlap, and the entire range of intensities for this concentration corresponds to very visible lines. Since a less developed test line corresponds to an improvement in the limit of detection for the competitive assay, it was unclear if the improvement we observed was primarily a result of this diminished line intensity or of the ATPS concentration. To determine this, we also investigated the use of the IL ATPS with a sandwich format assay.

### Detection of *E. coli*

Following the detection of Tf, we studied the detection of *E. coli* as a large biomarker to demonstrate improvement using an IL ATPS in a sandwich-format LFA, where diminishing of line intensities will have a negative impact on the detection limit. We performed experiments in a similar manner to the Tf tests. For this format, the top line still constituted the control line, indicating a valid test. However, the test line was comprised of primary antibodies specific to the target, rather than the target biomolecule itself. If the sample solution contains the target antigen, the antigen will bind antibodies on the GNPs, forming an antigen-antibody complex. This complex will then bind the immobilized antibodies on the test line, producing a visual red band. Conversely, if there is no target antigen, the antibody-antigen complex will not form and no test line will develop. Thus, for sandwich assays, two lines would indicate a positive test, while only one line would indicate a negative test. In this case, the limit of detection was defined as the lowest concentration of *E. coli* at which the test line was visible.

**Figure 5:**
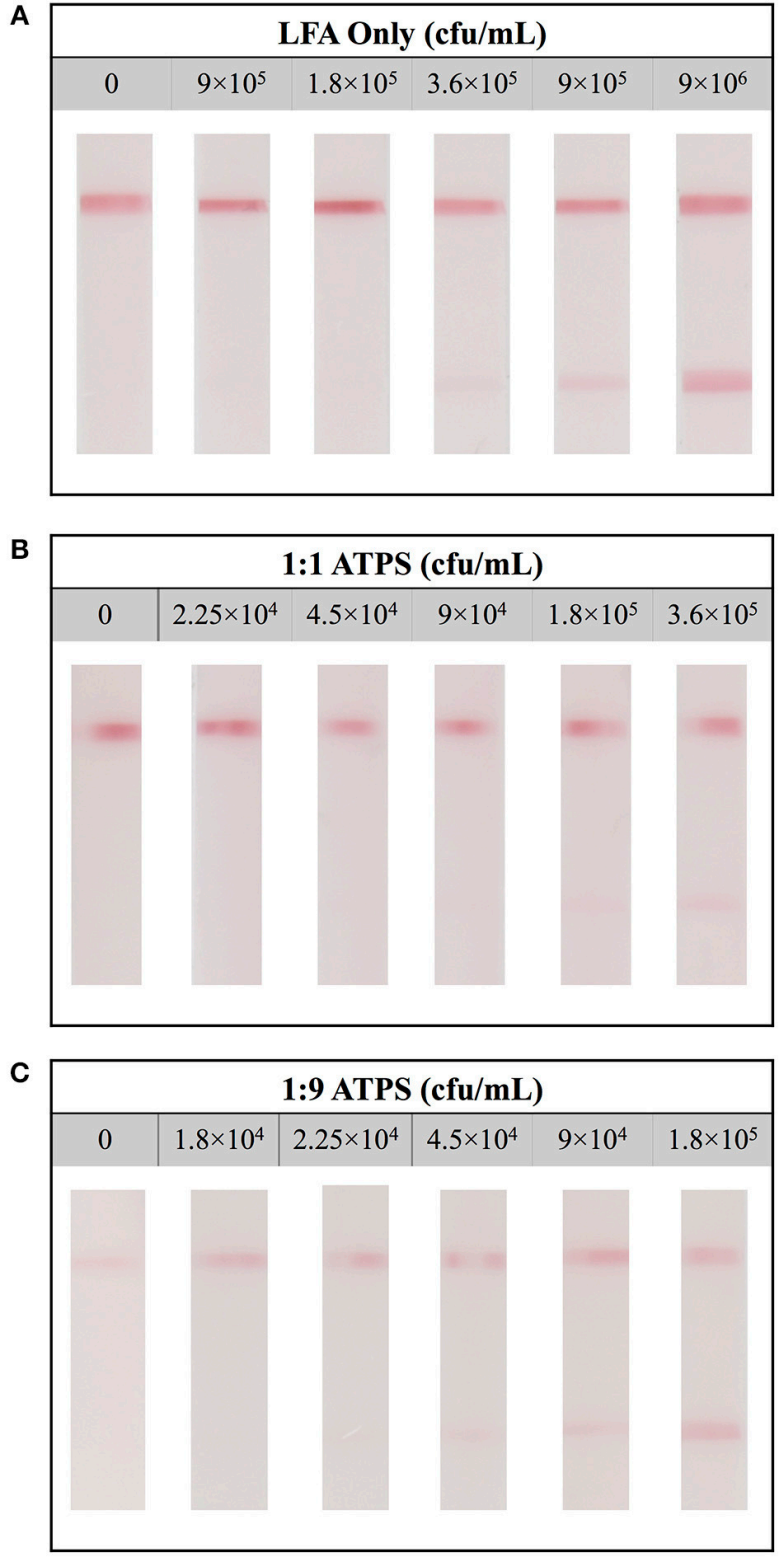
Limit of detection for LFA tests detecting for *Escherichia coli*. **(A)** Detection limit for the LFA-only test was found to be 3.6 × 10^5^ cfu/mL. **(B)** Detection limit for the LFA when combined with a 1:1 ATPS was 1.8 × 10^5^ cfu/mL and **(C)** detection limit for the LFA when combined with a 1:9 ATPS was 4.5 × 10^4^ cfu/mL, indicating 2-fold and 8-fold improvements, respectively.

**Figure 6:**
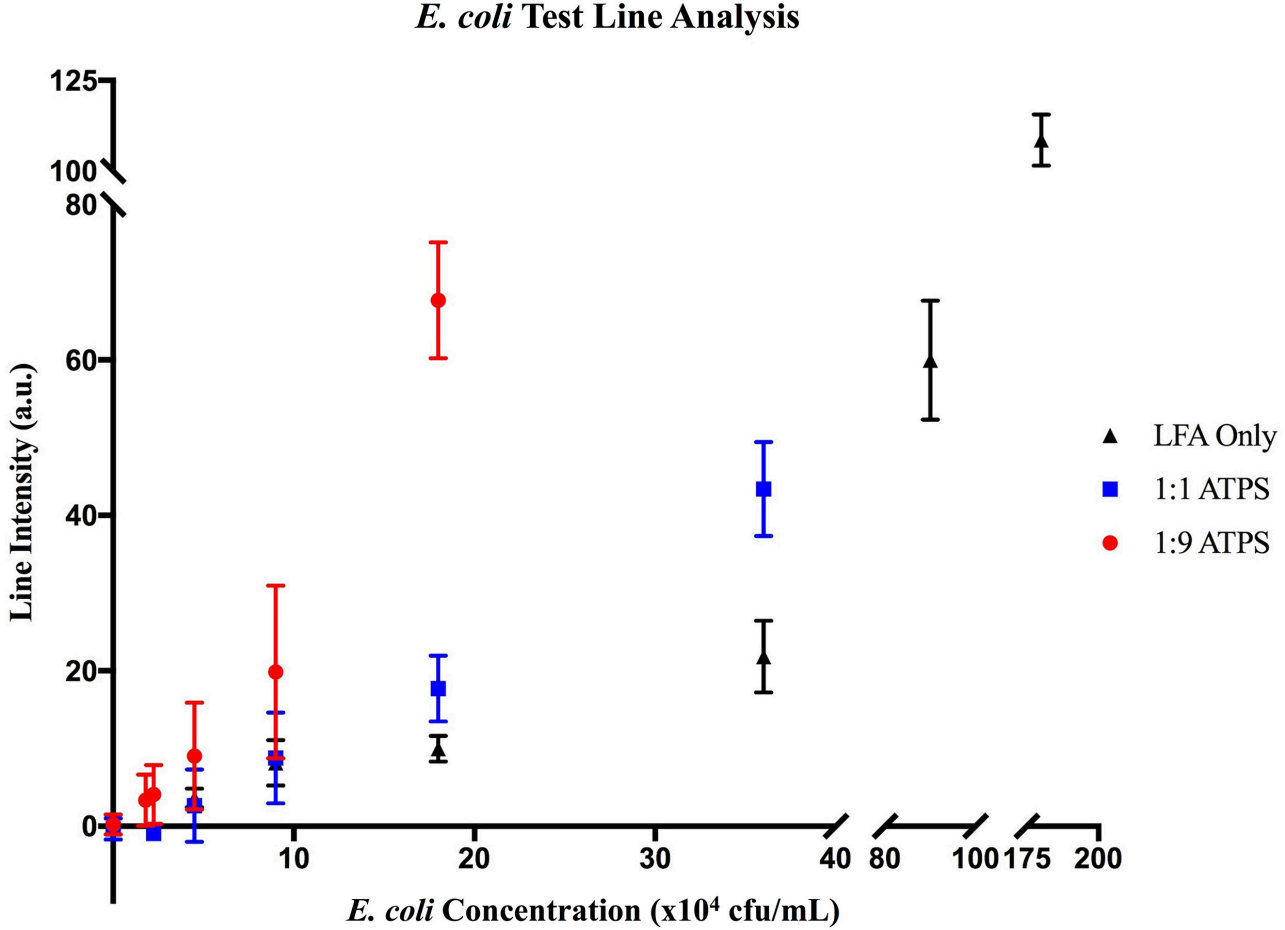
MATLAB analysis of *E. coli* LFA tests. Data for (▴) LFA-only tests without ATPS enhancement, (■) enhancement with a 1:1 ATPS, and (●) enhancement with a 1:9 ATPS. At each concentration, test line intensities in arbitrary units (a.u.) were greater for the 1:1 ATPS/LFA test than the LFA-only test and greater for the 1:9 ATPS/LFA test than the 1:1 ATPS/LFA test, indicating improved detection with more extreme volume ratios.

When testing with LFA only, at a high concentration of *E. coli* (1.8 × 10^6^ cfu/mL), two strong lines developed, indicating a true positive result. At a lower concentration (9 × 10^4^ cfu/mL), only one line formed, exhibiting a false negative result. These results suggest the limit of detection of *E. coli* using LFA only tests is 3.6 × 10^5^ cfu/mL (Figure 5A). Utilizing this analysis, the limits of detection for the 1:1 ATPS and 1:9 ATPS were determined to be 1.8 × 10^5^ cfu/mL (Figure 5B) and 4.5 × 10^4^ cfu/mL (Figure 5C), respectively, indicating 2-fold and 8-fold improvements in the limit of detection. While lighter line intensities than expected were still observed in these tests, the fact that improvement was still achieved in a sandwich LFA indicated that the concentration effect due to the IL ATPS was the dominant contributor to the improvement in detection limit. This analysis was confirmed via MATLAB analysis. As seen in Figure 6, test line intensity was improved with the application of more extreme volume ratio ATPSs, which corresponds with improvements in the detection limit of these tests.

## Discussion

Our group had previously demonstrated enhanced detection through the combination of LFA with ATPSs, specifically using polymer-salt and micellar systems; however, a primary handicap in the direct application of these ATPSs is their long phase separation time. As described previously, these systems can take hours to phase separate, which can limit their viability at the point-of-care. Our laboratory also demonstrated that the application of an ATPS to 3-D paper architecture drastically improves phase separation time (Pereira et al., 2015). However, this method requires modification to existing LFA devices to accommodate the enhancement technique. As the phase separation time of the IL ATPS for both 1:1 and 1:9 volume ratios were within 1 min and 5 min, this IL ATPS can be directly applied to existing LFA technologies, without necessitating modifications to the LFA. We hypothesize that this rapid phase separation speed can be due to several factors. This is further supported by the work of Gutowski et al., 2003; kosmotropic salts would increase the difference in dielectric constants between IL and water (Gutowski et al., 2003), promoting coalescing of similar domains in response to a high interfacial tension.

In addition to more rapid phase separation, the IL ATPS also displayed a greater degree of enhancement, specifically regarding the competitive LFA tests for Tf. While the degree of concentration for the 1:9 ATPS should be close to 10-fold, the improvement in the limit of detection was found to be 20-fold. We hypothesize this unexpected improvement is most likely due to the high ionic content of the system, which produces a screening effect that influences antibody-antigen binding. However, as use of the IL ATPS for the detection of *E. coli* still improved detection 8-fold, we determined that most of the enhancement seen for Tf is still a result of the ATPS concentration effect. The precise level of test line diminishment, and therefore the deviance from expected improvement, likely depends on the exact antibodies used in a particular assay. We do note that, in this study, both monoclonal and polyclonal antibodies for different antigens in different assay formats were successful, suggesting this system should still be widely applicable. Additionally, while the system does exhibit diminishing of line intensities, it should be noted that a primary motivation for use of this system would be to apply it to the partitioning of small biomarkers. These smaller biomarkers would generally require detection with the competitive LFA, as they typically do not contain many antigen binding sites required for use with the sandwich assay. Therefore, this screening effect only helps our system.

We envision the IL ATPS to be used for the detection of a wide range of proteins, with the ability to tailor the exact IL and salt system to accommodate the target of choice. To achieve this, we anticipate that a greater understanding of the phase separation mechanism of these IL-based ATPSs will be required. We also investigated the IL ATPS comprised of [Bmim][Cl] and potassium phosphate salt. Surprisingly, despite having similar components to our [Bmim][BF_4_] ATPS, this system displayed different phase separation behavior, consisting of an IL-rich top phase and a salt-rich bottom phase. While the exact mechanism of IL ATPS phase separation is not precisely understood, it is commonly believed that separation occurs due to the salting out effect of kosmotropic salts on the IL component. The degree of this salting out is likely a large factor in determining partitioning behavior and relative hydrophobicity/hydrophilicity of each phase. We found that this [Bmim][Cl] system yielded different gold nanoprobe partitioning behavior from the [Bmim][BF_4_] counterpart, with GNs partitioning extremely to the top IL-rich phase and DGNs partitioning extremely to the bottom salt-rich phase. While the ability to partition similar particles to different phases is promising, it is clear that a greater understanding of phase separation and partitioning behavior is needed to take full advantage of these capabilities.

## Conclusions

In summary, we successfully demonstrated the first use of an IL ATPS for the enhanced detection of biomarkers with the LFA. Specifically, a 20-fold improvement in the detection limit for transferrin was achieved utilizing a 1:9 volume ratio ATPS. This improvement can be seen as a combination of biomarker concentration, induced by the ATPS, as well as diminished test line intensity, likely due to screening effects from the ionic content of the ATPS. Despite the effects of the ATPS ionic content, an 8-fold improvement could still be achieved in the detection limit for *E. coli* using the sandwich-format LFA, where diminished test line intensities have a negative impact on the detection limit. Accordingly, most of the enhancement in the detection limit can be attributed to the preconcentration capability of the ATPS. Furthermore, this IL ATPS was found to phase separate very quickly, allowing for direct application to LFA without requiring modifications to existing LFA structure. While we demonstrated functionality using two biomarkers, we anticipate that further investigation into specific IL-salt pairings can enhance improvement for small biomarkers that would be difficult to concentrate otherwise. This combination of tunability and speed presents this system as a flexible, powerful enhancement tool for use with a wide variety of biomarkers and pathogens.

## Author contributions

MY, GE, and EY designed experiments, performed experiments, analyzed results, and drafted the manuscript. EL and JP performed experiments, analyzed results, and revised the manuscript. BW and DK designed experiments, analyzed results, and revised the manuscript.

### Conflict of interest statement

BW and DK are founders of the company Phase Diagnostics that intends on commercializing this core technology. They have financial interests in Phase Diagnostics, which has licensed this intellectual property from the UC Regents. The remaining authors declare that the research was conducted in the absence of any commercial or financial relationships that could be construed as a potential conflict of interest.
